# Policy challenges in the provision of COVID-19 border screening: evidence from eight countries

**DOI:** 10.1186/s12889-026-27355-8

**Published:** 2026-05-08

**Authors:** James Bates, Joshua R. Moon, Sibylle Gaisser, Anne Nikiforov, James G. Ryan, Choon Key Chekar, Robyn Meurant, Etienne Vignola-Gagné, Collins Iwuji, Erofili Grapsa, David Barbéra-Tomás, Enrique Meseguer, Gail Davey, Michael M. Hopkins

**Affiliations:** 1 Science Policy Research Unit (SPRU), University of Sussex Business School, Brighton, UK; 2 Ansbach University of Applied Sciences, Ansbach, Germany; 3 CIRCA Group Europe Ltd., Dublin, Ireland; 4 Lancaster Medical School, Lancaster University, Lancaster, UK; 5 ACT-IVD, Paris, France; 6 Elsevier, Science-Metrix, Montreal, Canada; 7 Africa Health Research Institute, KwaZulu-Natal, Durban, South Africa; 8 Brighton and Sussex Medical School, Brighton, UK; 9 INGENIO (CSIC-UPV), Universitat Politecnica de Valencia, Valencia, Spain; 10 Faculty of Law, Centre for Law, Medicine and Life Sciences, University of Cambridge, Cambridge, UK; 11 Technopolis Group, Brighton, UK

**Keywords:** COVID-19, Infectious disease screening, Cross-border health measures, Border management, Travel measures, International Health Regulations, Public healthcare services, Private healthcare services, Diagnostics industry

## Abstract

**Background:**

While border screening measures were widely adopted by countries during the COVID-19 pandemic, a lack of consensus on the utility of border screening created a gap in best practice for its implementation. As such, countries adopted a diversity of approaches, providing an opportunity to evaluate the configuration and evolution of border screening systems. The article addresses three questions: (i) how did countries configure their border screening systems for COVID-19? (ii) In what contexts did countries rely on public or private providers of these services? (iii) what do policies and narratives reveal about the perceived role of border screening in global public health? The article contributes to long-standing debates over the private sector’s role in public health and the perceived value of border screening measures.

**Methods:**

This article presents results from an international comparative study based on tracking the organisation of border screening in eight countries. Secondary data was collected between July 2021 – June 2022 from official government websites and policy publications, private sector sources where relevant, and trusted media sources in each study country. The countries included are Australia, Canada, Germany, Ireland, South Africa, South Korea, Spain, and the United Kingdom.

**Results:**

All study countries used private provision for pre-departure diagnostic testing for international travellers. In contrast, screening of arriving travellers was more diverse. Countries that opted for private sector post-arrival screening saw governance challenges around accreditation and monitoring of providers, while public service provision saw challenges in capacity and high resource costs. Travel was often framed as a ‘luxury,’ allowing states to shift responsibility for obtaining tests onto individuals; especially in the context of individuals travelling from low income to high income countries.

**Conclusions:**

The different approaches countries followed for screening of departing and incoming travellers suggests wealthy countries were more oriented towards defending their populations against disease importation, rather protecting the international community from disease exportation. These findings provide an opportunity to reflect on the purpose and implementation of border screening. We emphasise a need for further discussion on the efficacy of border screening from both perspectives, given the tendency for countries to rely on these measures.

## Background

The COVID-19 pandemic led to widespread uptake of border screening measures implemented with the aim of reducing the international spread of infection [1, 2]. In dynamic circumstances national authorities sought to balance public health protection with the need to engage in continued international travel and trade. There has been much debate over the efficacy of border screening measures during public health emergencies [3, 4]. Yet there remains limited evidence as to the kinds of measures used in different contexts during the COVID-19 pandemic [5], and as such, a continued lack of consensus on the challenges of configuring, organising, and delivering public health measures at national borders.

Grépin et al. [6] found that travel bans and border screening have a limited impact on the overall course of outbreaks, delaying epidemic onset by 1–3 weeks, depending on the timing of policy introduction. However, the evidence reviewed by Grépin et al. varied widely in their efficacy assessments, model validation, and accounting for other public health interventions. Another relevant review by Mouchtouri et al. [7], similarly found that many studies could not record a substantial reduction in disease incidence, with rates of successful case identification being extremely low. However, they did identify the presence of other potentially beneficial contaminant effects of screening at the border with impacts that may be difficult to concretely assess, including raising awareness and educating travellers, discouraging travelling of ill persons, and other benefits of maintaining flights to/from affected areas [7]. The lack of consensus in evidence for border screening’s efficacy, along with international norms against such measures [3], contributed to the World Health Organisation’s (WHO’s) initial recommendations against implementing “any travel or trade restriction” [8] in February 2020.

Despite this, two weeks after WHO recognised COVID-19 as a pandemic, almost 100 countries had implemented travel bans, quarantines, or screening for travellers incoming from China [9], against WHO’s recommendations [8]. Regardless of the efficacy of border screening, countries have implemented these measures in previous epidemics and will likely continue to do so as part of wider national preparedness and response. This paper does not advocate for or against border screening as a public health policy, but instead acknowledges that these measures can and will be deployed, while identifying the challenges associated with different configurations of these systems. By examining border screening in eight countries, we aid understanding of the trade-offs between different testing configurations before, at, or after crossing a national border. We are thus address the following questions: how did countries configure their border screening systems for COVID-19? In what contexts did countries rely on public or private providers of these services? What do policies and narratives reveal about the perceived role of border screening in global public health?

### International travel and trade in public health emergencies

Increasing globalisation and movement of individuals across borders raises the cost and complexity of managing infectious disease threats as potentially infectious individuals may travel through multiple countries with distinct health systems [10, 11]. The international nature of public health emergencies thus requires international cooperation and mobilisation of resources at the global level. The introduction of travel and trade restrictions is a common practice for many states in the event of an infectious disease outbreak. However, these restrictions come at an economic cost, as lost trade may impact the restricting and the restricted nation.

Challenges arising from disease controls at the border have long been recognised, with the first international sanitary conference being organised in 1851 to coordinate international action on Cholera, and to tackle a tension between open international trade routes and a need to protect public health. This began what David Fidler (2005) [12] terms the ‘classical regime’ of international health which rests on principles of state notification of disease and limiting public health policies which harm international trade. These principles balance a need to be globally responsive to public health threats whilst maintaining an openness to trade relationships to limit economic harm.

Importantly, the classical regime’s two principles of early notification and limited use of measures that restrict travel and trade are built upon a need to maintain this balance of public health and economic openness between states. The revisions made to the International Health Regulations (IHRs) in 2005 made this state-to-state compact broader by shifting scope from specific diseases to any public health event that fulfils specific criteria. This means that international cooperation in health depends upon mutual trust that states with an outbreak of pandemic-potential will notify others early and transparently, and that economic partner states will not implement excessive travel and trade measures in response.

Consensus against travel and trade measures, however, is not limited to states’ economic needs or terms; the argument made within the public health domain and within broader academic discourse is one of equity and access. Regarding equity, consideration is given as to which countries are most likely to notify a potential Public Health Emergency of International Concern (PHEIC); The expectation is that likely sources of cross-border spread will tend to be often poorer, often postcolonial, often global south states with limited health system capacity to contain a novel infectious disease [13]. Further, those countries most likely to implement travel and trade restrictions are often richer, often former imperial, and often global north states, with this dynamic reinforcing global inequity with poorer states being repeatedly harmed while global north states seek to contain infectious diseases ‘over there’ [14].

In terms of access, travel and trade restrictions can also impede the entry and exit of public health personnel and resources, particularly when aid is needed to support states’ responses, which results in a less effective response [15]. This is particularly the case for medical materials which require sufficient cold-chain capabilities to maintain viability in transit, as seen with COVID-19 where the delivery of near-expired vaccine doses created a need to both accept, store, and distribute vaccines urgently with minimal inhibition to transport [16]. In both cases, travel and trade restrictions are seen as a hindrance to both public health and foreign policy objectives.

This issue was further highlighted in the revisions of the IHRs passed by the Seventy-seventh World Health Assembly [17] which explicitly revisit this issue. In making changes to Article 18 (referring to the issuance of temporary recommendations), a new paragraph (para. 3) now notes that recommendations should “take into account the need to:

- Facilitate international travel, particularly of health and care workers […]
- Maintain international supply chains, including for relevant health products and food supplies”

Similarly, the Zero Draft of the proposed Pandemic Accord [18] refers to parties’ commitment “not to impose regulations that unduly interfere with the trade in, or of, pharmaceutical raw materials and ingredients, mindful of the need for unhindered access to pandemic-related products” [p.13]. These changes across two international-level negotiations clearly emphasise a need to provide open travel channels for essential medical and support personnel and resources.

Despite being the consensus for over 150 years, this state-to-state compact, where limited border measures and early notification are expected, has been flouted multiple times already this century. In 2003, China’s reluctance to report SARS as it was emerging was met with criticism [19, 20], while the peak of the outbreak saw 110 countries place some kind of travel restriction on China. The 2009 H1N1 Influenza pandemic saw prompt reporting from Mexican authorities, but this was followed by 20 countries placing bans on pork and pork products coming from Mexico [21–23]. In 2014, during the West African Ebola outbreak there were delays in reporting due to a lack of infrastructure and surveillance capacity in-country and a spate of travel bans for many African states, including some countries which had no cases of Ebola [24, 25]. At its core, the espoused norm which sits around the classical regime and which was codified in the IHRs has rarely — if ever — been upheld by states [26]. This is also true of the COVID-19 pandemic where delays and a lack of transparency in reporting were met with wide-spread travel restrictions [9, 27, 28].

The trade-off codified within the ‘classical regime’ of international health, that seeks to limit impacts on travel and trade due to outbreaks in return for early notification from states, was highlighted again in the early days of the COVID-19 pandemic. Initial delays in reporting initial cases of a pneumonia of unknown origin (later to be identified as SARS-CoV-2) in Wuhan, as well as perceived delays in response to WHO’s issued Article 10 request [27, 28], created a perception that Chinese authorities may have been downplaying the extent and severity of the outbreak [29]. However, as the reality of COVID-19 became clear, a spate of travel and trade restrictions were instated, and, by April 3rd 2020, 96 countries had imposed some kind of border measure [9]. The state-state compact of the classical regime, codified into international law by the IHRs, had been broken yet again.

International cooperation for combatting COVID-19 was advised by multiple transnational organisations such as the UN [30], WEF [31], WHO [32], and in academia [26]. Many early commentaries condemned travel restrictions as discriminatory [33], breaching international law [34, 35] and negatively impacting the COVID-19 response [36, 37], once again arguing that the restrictions are bad for public health and harm economies. Regardless of whether or not border measures are legal, normatively acceptable, or even effective, it is clear that travel and trade restrictions are a common feature of state responses to PHEICs. It is therefore imperative to better understand the motivations, limitations, and expectations surrounding their use.

### Border screening as a sub-set of travel and trade measures

**Fig. 1:**
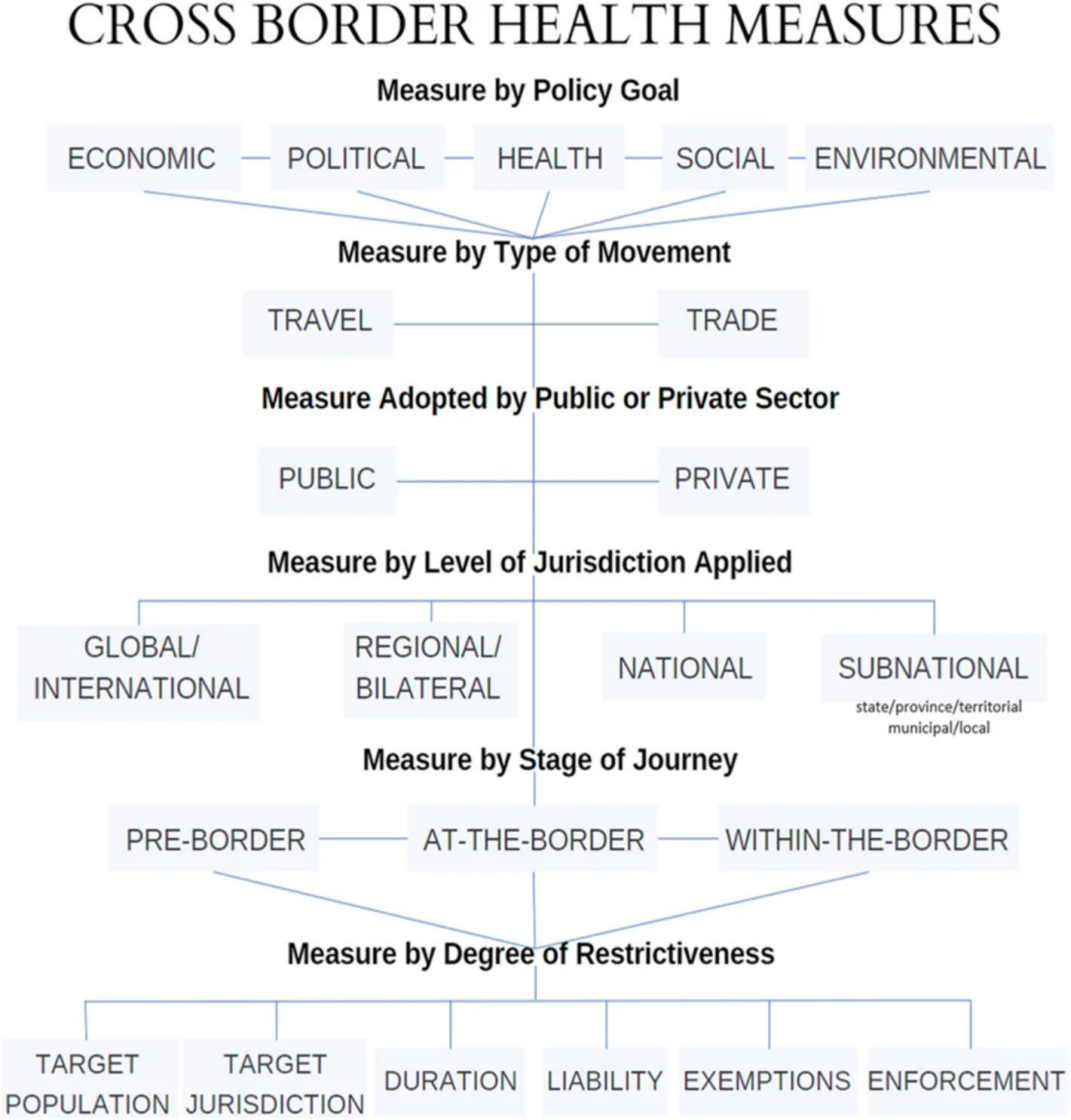
Typology of cross-border health measures. Source: Reproduced from Lee et al. [38]. under the CC BY 4.0 License: https://creativecommons.org/by/4.0/

Travel and trade measures are not a single policy prescription but a suite of potential measures that prohibit or encourage travel & trade to differing extents. While some countries closed their borders outright, others closed them to specific populations, and others implemented varying degrees of visa requirements. To parse this distinction, Lee et al. [38] developed a typology of such measures (Fig. 1) categorised by policy goal, type of movement, policy adopter, jurisdiction, stage of journey, and restrictiveness.

In response to COVID-19, as noted by Lee et al. [38], “*few jurisdictions [were] actually closing their borders or banning travel … a variety of measures have been applied and lifted over time for controlling who travels and under specific conditions*” (page 2). Importantly, Lee et al.*’s* typology does not refer explicitly to *what* measure is implemented, be it symptomatic or diagnostic screening, isolation or quarantine; Lee et al*.* are primarily interested in the character of the measure.

Border screening is a core capacity for states, as defined by the 2005 revised International Health Regulations (IHRs) [39]. Annex 1B of the IHRs lays out the core capacities for states with respect to designated airports, ports, and ground crossings. Paragraph 2 points (d) and (f) of this annex require that, when responding to events that may constitute a PHEIC, states should have the capacity “(d) *to provide for the assessment and, if required, quarantine of suspect travellers [and] … (f) to apply entry or exit controls for arriving and departing travellers*” [39] [page 41]. This implies a level of symptomatic or diagnostic assessment and screening at borders, at least for travellers suspected of infection, and the ability to not only quarantine but apply entry and exit controls on confirmed cases.

Border screening relies on three components: 1) availability of appropriate symptomatic or testing capabilities at borders; 2) sufficient capacity to test in volumes equivalent to border transit volume; and 3) policies, capacities, and enforcement for care, treatment, and isolation of positive cases (IHR Annex I, Part B, Sect. " Methods"). Border screening of travellers from particular affected regions has been implemented for various infectious diseases, before and after the COVID-19 pandemic. For example, routine screening programmes exist for diseases including tuberculosis [40], and mosquito-borne diseases such as Dengue fever, Chikungunya, and zika virus [41]. Other screening programmes may not include diagnostic testing, but include certifications of vaccination (as with Polio or Measles), or through monitoring incursion of disease vectors such as mosquitos across national borders [42]. Following the COVID-19 pandemic, certain countries reintroduced border screening for Mpox disease, such as South Africa in June 2024 [43].

In January of 2020, WHO’s advice when declaring a PHEIC explicitly stated that “*it is generally considered that entry screening offers little benefit, while requiring considerable resources*” [44]. However, the same advice also stated that: “*As provided by the International Health Regulations (2005) (IHR), countries should ensure that: routine measures, trained staff, appropriate space and stockpile of adequate equipment in place at points of entry for assessing and managing ill travellers detected before travel, on board conveyances (such as planes and ships) and on arrival at points of entry*” [44]. The same declaration also noted a recommendation for China to “*conduct exit screening at international airports and ports in the affected provinces, with the aims early detection of symptomatic travelers [sic]”* [44]*.* Importantly, the recommendation around border screening advanced here is not an externally imposed travel or trade restriction designed in national self-defence, but an altruistic use of resources to prevent international spread by an affected country.

These two applications of border screening (defensive or altruistic) and their relative priority when screening is implemented by states is important when we consider the norms that underpin the classical regime, and general trends in international travel. A core assumption of the classical regime is that international spread of infection is the responsibility of the affected state, and thus that states should notify early, in return for minimised barriers to travel and trade; in essence, the focus is on affected states limiting the export of cases rather than unaffected states limiting the importation of cases. Statistics for 2020’s air traffic shows that 5 of the 10 busiest airports (by number of international passengers) were European, two are high-income Asian countries, and three are international hubs in the Middle East [45]. If the norm within the classical regime is to reduce the international spread of infectious disease, it seems incumbent on countries with the highest levels of traffic to be take responsible measures to limit their *exportation* of infected cases rather than simply being concerned about the importation of such cases into their territories. As our findings will show, however, there seems to be more prioritisation of direct state resourcing and provision of screening to control case *importation* over exportation [46].

The implementation of border screening, therefore, is of central concern to national states looking to limit the spread of COVID-19. However, if nations diverge in strategy and operational implementation, this can create barriers to international cooperation on cross-border health management [47–49]. All of our study countries quickly replaced COVID-19 symptomatic screening with diagnostic tests once these were available. As such, our empirical focus is on disease-specific testing, rather than general measures such as temperature screening. This paper thus defines border screening explicitly as the use and/or provision of *diagnostic tests* for individuals travelling across national borders either pre-departure, at-the-border, or post-arrival.

### Privately provided public health & the ‘luxury’ of travel

As the global COVID-19 pandemic progressed, states increasingly delegated activities to private providers to contribute additional screening capacity in areas where public capacity may be insufficient [50–52]. This increase in private provision, however, also brings concerns over the degree to which public health outcomes are prioritised. Frequent criticisms include misleading marketing practices that can lead to uninformed health decisions, concerns about the privacy and security of genetic data, and the lack of professional counselling to support consumers in understanding test results [53–55]. This had the potential to cause significant harm and exemplifies the need for regulatory oversight and informed consent [56].

As well as concerns over companies providing direct-to-consumer testing services, there is a long-standing debate on the private sector’s role in delivering laboratory services with a clear public health goal [57, 58]. There have been concerns that actors with commercial incentives will have other objectives besides delivering optimal public health outcomes. For example, the increasing privatisation of National Health Service (NHS) activities in the UK has raised concerns over the quality of private service delivery [59]. Furthermore, critics of the ‘privatisation’ of NHS services also recognised aspects of the UK's pandemic response have contributed further to this trend [60]. There was an explicit emphasis by the UK government to build an effective private market for diagnostic testing, as a key pillar of the COVID-19 response [61]. At the same time, resource constraints in the public sector may make operating border screening through the public sector difficult when there is simultaneously high demand for community and hospital testing [62].

In addition to these more general concerns surrounding a commodification of public health, there are sharper IHR-related issues with respect to the ‘luxury’ framing of travel & screening. Article 40, paragraph 1 of the IHRs states that:

*“no *charge* shall be made by a State Party pursuant to these Regulations for the following measures for the protection of public health:*

- *(a)* *Any *medical* examination provided for in these Regulations, or any supplementary examination which may be required by that State Party to ascertain the health status of the traveller examined;*
  […]
- *(c)* *Appropriate isolation or quarantine requirements of travellers.”*

This would suggest that, in the case of private provision of testing for border screening purposes, the requirement for travellers to pay for tests required by States Parties whilst travelling may not comply with international law. However, paragraph 2 of the same article states that “States Parties may charge for health measures other than those referred to in paragraph 1 of this article, including those *primarily for the benefit of the traveller*” (emphasis added).

The degree to which the private sector delivers border screening is of particular interest here, then, because of the extent to which travel was framed as a ‘luxury’ activity during the COVID-19 pandemic, and thus primarily of benefit to the individual traveller [63–65]. Indeed, there are considerable opportunities for profitable business in the context of a ‘luxury’ framing of international travel [66], as indicated by the large number of new market entrants and rapid growth of this market for diagnostic tests [67, 68]. This framing of travel as a luxury places a public health responsibility (and thus responsibility for cross-border spread) on individuals, rather than on the state. This allowed states to defer financial responsibility, requiring travellers to pay for their own individual testing. Similarly, this allowed the private sector to provide services for the screening of travellers on a consumer basis, reducing burden on public testing provision.

The importance of border screening as a public health measure, [3, 69, 70] and the implications of private provision in support of this ‘luxury’ framing thus motivates a detailed analysis of how border screening was charactered and implemented during the COVID-19 pandemic. Specifically, analytical focus in this article is placed on where and why screening activities were delegated to the private sector and what this suggests about intentions and framings of responsibilities for cross-border spread.

## Methods

This article draws on multiple case studies, enabling a comparison of approaches to a particular issue and facilitates the generation of theories based on real-world data [71–73]. As addressed by Gilson [74], this method aims to produce analytic generalisation, rather than drawing conclusions to be statistically proven or applicable to wider contexts. The desired output is “general conclusions that, although derived from a limited number of particular experiences, provide theoretical insights that can be put forward for consideration, and testing, in other, similar situations” [73] [page 3]. This approach is most appropriate as our aim is to identify narratives and generate insights into challenges associated with different configurations of border screening internationally.

The countries included are Australia, Canada, Germany, Ireland, South Africa, South Korea, Spain, and the United Kingdom (hereafter the ‘study countries’). The countries studied were selected as part of a wider project [75], intended to provide a diverse set of policy responses from a geographically diverse set of countries spanning a broad range of mortality rates from COVID-19 (when selected in mid-2020). Secondary data was systematically collected from publicly available information sources on the provision of public and/or private COVID-19 testing services for travellers by the authors, being researchers familiar with the local contexts studied. Discussion of temperature screening at the border is excluded from our analysis as this practice had been reduced in our study countries following a growing consensus of the questionable efficacy of this type of screening [76, 77], and a rapid evidence review delivered by Public Health England [78]. Moreover, additions to IHR Core Capacities in the updated IHRs Annex 1, part B, Sect. " Methods" now refer explicitly to laboratories and sample analysis as core capacities at relevant points of entry. This article is therefore primarily concerned with the provision of PCR and rapid-antigen tests (RATs) as part of broader screening and surveillance policies within our study countries.

Secondary data collection consisted of information from official government websites and policy publications regarding guidance and arrangements, private sector sources where relevant, as well as trusted media sources in each study country. Further data was also collected from the World Bank Development Indicators database [79] to inform aspects contributing to the design of border systems, such as air passenger travel numbers for certain reasons. In the event that official sources do not provide the necessary data, primary sources have been used, such as questionnaires via e-mail with health authorities responsible for border control. Information was organised into broad categories based on elements of each system, the issues noted to be arising in each context, and any policy responses taken to address emerging issues. Regular discussion took place amongst the authors to agree questions, answers and analysis, together with central compilation of results (by authors JB, SG, JM, AN) to ensure consistency. This data collection sought to cover developments from 1 st August 2020–1 st June 2022, with data gathering conducted between July 2021 – June 2022.

The analysis provided does not compare the stringency of border measures internationally or their impact: measures in the eight study countries have changed often, making it challenging to analyse stringency in a rigorous manner. The analysis provided in this article also does not assume that border screening is an inherently effective intervention, nor does it aim to directly compare the efficacy of border screening policies internationally, which it is recognised comes with many caveats [80, 81]. Rather, we provide qualitative insights into problematic issues raised by different configurations of public or private border screening, as well as any policy measures adopted to address these issues.

## Results

Policies for border screening for departing travellers were relatively similar across the eight study countries, and so these findings can be summarised relatively briefly in 3.1 below. By contrast, we observe considerable variation in the organisation of border screening systems for arriving travellers particularly in 2020. However, over time, and following the introduction widespread vaccination against COVID-19 and RATs in 2021, we see greater convergence in boarder screening policies in the study countries. Sect. "Post-arrival screening August 2020 – October 2021" and "Post-arrival Testing: October 2021 onwards" reflect these changing practices and present our findings for border screening of arriving travellers in two distinct time periods: from 1 st August 2020 to 1 st October 2021; and from 1 st October 2021 to 1 st June 2022. This cut off was chosen as it is the date by which half our study countries had achieved the WHO target of 70% fully vaccinated population. This was generally also the point by which more relaxed measures were implemented in those study countries that had the most stringent controls in place (South Korea and Australia).

### Pre-departure screening: public health rationales and state-centrism of public health

All eight study countries were observed to have delegated the provision of pre-departure screening services entirely to the private sector, with travellers required to pay a fee to access screening services at their own expense when travelling abroad. The consensus observed here in specifically excluding pre-departure border screening activity from the scope of public health laboratories, may reflect the state-centrism of public health provision [82]. This is in contrast with the recognised need for global cooperation in cross-border health management during a global pandemic, [26, 83]. Cooperation and consensus in organisation may have been hindered by the continuing debate over the efficacy of border measures [7, 84–86].

A second point of note is that in the study countries where pre-departure testing was mandated, the requirements came from the destination country, rather than countries of origin. During the COVID-19 crisis, countries often set variable requirements for pre-departure testing dependent upon national need and varying by country of origin [87]. The UK, for example, instigated different rules for countries based on their epidemiological context, which was dynamically categorised as ‘green’, ‘amber’, or ‘red.’ These designations changed often and not always due to publicly available epidemiological information, leading to disruption for travellers entering and exiting the UK [88, 89]. By having varying requirements for different countries of origin, we again see a conflict between public health priorities and economic and foreign policy priorities for decision-making, highlighting state-centred policymaking that appears to be at odds with the ‘classical regime’s desire for cooperation.

In addition, the implication of these policy decisions is that the priority for border screening is to prevent the *importation* of cases into the country. The setting of pre-departure requirements by the destination country emphasises this because the destination country receives the benefit (fewer imported cases) while the travellers from country of origin accepts the cost (of testing provision) and citizens of the origin country accept the risk (cases remain in-country). Subsequent delegation of screening provision to private providers by the country of origin reveals this priority because it puts at arm’s length implementation of a policy that protects other countries, while maintaining public provision of screening of inbound travellers as this addresses their national public health priority. This runs counter to the narrative within the IHRs and the WHO Emergency Committee recommendations [44], discussed in Sect. " Background", which place emphasis on border screening for preventing case *exportation* as part of international cooperation.

### Post-arrival screening August 2020 – October 2021

Table 1 provides a brief description of each of the study countries’ post-arrival screening systems, placed according to the degree to which screening was provided by the private sector and whether travellers pay for the test directly. In some countries, changes in screening policy relating to renumeration and modes of screening provision were frequently observed at the national and state levels, and illustrative dates are provided for our study period. However, it is not our purpose to present a complete timeline of policy development, but rather to demonstrate that policy priorities to provide screening at the border through public sources can change for multiple reasons including epidemiology, vaccination rates, and available public/private capacity.

**Table 1:** Quadrant to show the variation internationally in arrangements of post-arrival testing in 2020 and early 2021. The details of these policies in the table provide examples of how systems can be specified, rather than aiming to provide a comprehensive timeline of policy changes. Notably, a) after October 2021 more countries (namely the UK, South Korea, and Australia) converted to use of rapid antigen testing post-arrival. b) not all countries (Ireland.& Canada) implemented use of RATs for post arrival screening, preferring to remain using PCR testing until the end of border screening requirements entirely

		Do travellers pay for post-arrival screening?	
		Free	Paid
Test provider	Public Sector	South Korea (PCR): From 22nd March 2020 [90], all travellers and returnees were subjected to a mandatory test for COVID-19 at public facilities and 14-day quarantine at government facilities or in their own homes regardless of test results Australia (PCR): From 28th March 2020 [91], all returning travellers were required to undertake 14 days of quarantine in a designated facility. Here, travellers were tested regardless of symptoms at days 2, 11, and 14, at which point they could leave hotel quarantine. This testing was organised through by the public sector, which also utilised private sector lab capacity Spain (RAT): From 17th November 2020 [92] until end of border measures on October 21 2022 [93], travellers without adequate pre-departure test/vaccination documentation, or who displayed symptoms during a visual ‘health check’ by border authorities, had to undertake a free on-arrival test at airports was sometimes required during which determined the requirement to quarantine. Spain appeared to not use any PCR testing system post-arrival Ireland (PCR): From 29th November 2020 [94], Post arrival quarantine requirements could be ended early by using PCR tests on day five, but only for passengers from some EU-Member States. Screening was available for free at Dublin airport	UK: Scotland & Wales (PCR): From 15th December 2020 [95], post-arrival screening requirements were introduced, where travellers could test during quarantine to be released early. While England and Northern Ireland enabled a private market to deliver this, in Scotland and Wales, only public system (NHS) tests were available to be purchased at a fixed price, sold through a portal managed by a company called ‘Corporate Travel Management” South Africa (RAT): Limited post-arrival screening system, there was no requirement for post-arrival screening. However from 29th March 2021 [96], on-arrival antigen tests at the airport were required only for those without valid pre-departure test documentation, or those who developed symptoms during travel. Tests were to be paid for by the traveller and the result would determine requirement to enter quarantine. Since tests appear to have been provided by the airport, the individual did not have to seek out a private provider to do so
	Private Sector	Canada (PCR): 22nd Feb 2021–9th August 2021 [97]. Arriving travellers were provided with a PCR home-test kit for them to complete during home-quarantine. The kit included instructions to register online with a private provider who supervised sample collection remotely and completed the test at their laboratories. Random airport arrival testing was also completed by border authorities. All border screening was reimbursed by the Canadian Government. After August 9th 2021, fully vaccinated International travellers no longer needed to conduct post-arrival screening [97] Germany (PCR): From 27th July 2020 [98], post-arrival screening requirements were introduced for arriving travellers from high-risk areas. In Germany, arrival testing was largely carried out at centres operated by private companies. Travellers did not have to pay for the cost of tests. By 15 October 2020, testing on day 5 could be sought out to end quarantine early	Germany (PCR): From 15th September 2020 [99], ‘low-risk’ travellers were required to pay for arrival tests, whereas higher risk travellers had costs reimbursed until 16th December 2020 [100]. Organisation of provision was delivered at the state level, and changed multiple times in each throughout the remainder of the pandemic, depending on shifting epidemiology and vaccination rates. A notable change occurred in October 2021 when the German government ceased provision of free screening for unvaccinated individuals UK England & Northern Ireland (PCR): From 15 December 2020 [101], England enables a private market for post-arrival screening companies. Commercial providers were listed on a government website, searchable by location, test type and price. Individuals could also purchase public sector NHS tests; through a private booking system also used in the other devolved administrations (Scotland and Wales) Germany (RAT): From January – May 2021 [102, 103]: As part of Germany’s “two-test strategy” RATs could be used as on-arrival tests for high-risk travellers. Furthermore, RATs could be used to exit quarantine on Day 5 was enabled for arriving travellers from low-risk areas. Regulations were continually updated in response to epidemiological conditions, however this exit from quarantine testing option remained throughout. Tests could be received from commercial test centres, which were associated with concerns over low quality as authorities struggled to quality control. From 12th of May 2021 [104], quarantine (and subsequent testing) requirements began to be lifted for lower risk countries

Some study countries (Spain, South Africa, and in a limited fashion, Canada and Germany) conducted on-arrival screening at airports, for individuals who for any reason lacked correct pre-departure test documents, or individuals who appear to be displaying COVID-19 symptoms. More commonly (in Australia, Canada Germany, Ireland, South Korea, and the UK), post-arrival screening regimes consisted of a requirement for PCR tests or RATs on specific days during a mandatory quarantine period, following arrival, that individuals completed either at home or in designated facilities.

#### Public provision

Freely provided post-arrival PCR screening was under the remit of public health services in Australia, Ireland, and South Korea (Table 1). Each system experienced organisational challenges associated with delivering this public testing provision [62], with those countries that did not allow self-testing or unsupervised self-sample collection experiencing additional challenges with implementation.

Ireland required arriving travellers to attend in-person clinics that were also used for public health symptomatic PCR testing [105]. However, Ireland’s Health Information and Quality Authority (HIQA) recognised low adherence to testing requirements, with compliance by travellers perhaps as low as 35% [106]. Issues included easing access to test centres, inadequate communication that testing was free and mandatory [105, 107], and a need for better data collection [105]. Australia, similarly, had stringent requirements for screening with extensive mandatory hotel quarantine for all travellers. Samples were collected and delivered as part of the public testing system for symptomatic cases [108], which utilised private provider laboratories at times to increasing screening capacity. Criticisms were similarly related to the restriction of freedoms for travellers [109, 110], although a national review of hotel quarantine in Australia only covered testing in passing [91]. Unlike Ireland, issues of low uptake were not reported.

South Korea’s approach distinguished between foreign visitors without a permanent residence and Korean nationals. While foreign visitors completed hotel quarantine, those with a residence used a mandatory paid ‘quarantine’ transport (designated carriages on trains, special buses, or taxis) to shuttle individuals from airports to a public PCR testing site, and then to their home where they waited for results [111]. The result of this test would determine if they were required to isolate in a government-managed quarantine facility if positive or continue to isolate at home if negative [112].

Delivery of diagnostic tests to home-quarantined travellers added to the organisational complexity of border screening systems. Australia, Ireland and South Korea had not enabled self-sample collection or self-tests for much of the period where travel restrictions were in place, which would have improved access to testing for travellers. Experts in these countries expressed concern over self-test use, and the possibility of false negative results, due to inadequate sample quality [113–116]. However the benefits to relieving the burden of workload on healthcare professionals and easing access to tests were also recognised [117].

#### Private provision

Provision of screening by private providers varies in this period by whether individuals were required to pay for their test themselves. In Germany, post-arrival screening was largely carried out at centres operated by private companies [118], while the reimbursement of the costs of testing varied [119–122]. *By* the 16th December 2020 screening was no longer provided for free and higher-risk travellers also had to pay for arrival tests [123]. Canada involved contracting private diagnostics companies to deliver an online system of remote supervision of swabbing overseen by healthcare professionals [124, 125], the cost of which was reimbursed by the federal government [126]. Private provision in this case was associated with a decrease in quality of screening services [127–129], including 17% of results being returned after the 14-day quarantine period and unqualified healthcare professionals being hired to supervise sample collection [130].

The UK provides a comparator where, from 11th March 2021, England and Northern Ireland instead enabled an open privatised market where travellers choose providers and pay for tests themselves [131, 132]. The GOV.UK website provided travellers with a list of approved private providers of day 2, 5 and 8 testing, filtered for region, type of sample collection, or test price [133]. Initially, various media sources and consumer watchdogs reported travellers were being misled over the prices of tests available from these companies [134–136]. This prompted the Health Secretary to commission an investigation by the UK Competition & Markets Authority (CMA) [68, 137–139] which cited problems of ‘bait-pricing’, ‘drip-pricing’ & ‘price gouging’ and corroborated media reports of inadequate timeframes for delivery of tests and results [140–146]. Eventually, there was widespread scrutiny surrounding England’s PCR travel testing market [147–149] and the UK Department of Health & Social Care introduced a 2-strike system, wherein companies were warned and then delisted if found to be advertising misleading prices again [150, 151].

In a departure from PCR testing, South Africa required on-arrival RATs at airports if a traveller had incorrect pre-departure test documentation or exhibited symptoms, with the result determining entry into mandatory quarantine in a government facility [152]. Screening was primarily provided by the private sector, with individuals being required to pay for the costs of testing themselves. Private screening services in South Africa were common and also had a role in supporting the public sector in providing PCR testing for symptomatic individuals in the early stages of the COVID-19 response [153]. However, it was reported that private providers offered a much faster turnaround than the strained public system [154], and more broadly, this two-tier system leads to suboptimal health outcomes for many South Africans [155–157]. The use of private providers in these cases, then, indicates a series of choices made about where to best allocate limited public screening capacity and resources.

### Post-arrival testing: October 2021 onwards

During the initial response to the pandemic, and particularly in the first phase of our data collection outlined above, there were strong concerns raised by various expert groups around the use of RATs in screening asymptomatic individuals. RATs’ lower sensitivity compared with PCR tests [158] raised concerns about ‘missing’ cases that were in early-stage infection and likely to become infectious in the days after arrival [159]. However, despite this concern such tests were eventually widely used for such purposes (including screening at the border, as well as accessing public events) due to their associated benefits including lower cost, improved access to testing, and cases found that otherwise may not have come forward for PCR testing. Table 2 summarises the countries’ systems in this period.

**Table 2:** Quadrant to show the variation internationally in arrangements of post-arrival testing, only including developments post 1 st October 2021. Testing requirements in some cases ended prior to 1 st October 2021 and therefore are not included in this table, namely in Canada (August 2021) and in Germany (May 2021), as detailed in Table 1

		Do travellers pay for post-arrival screening?	
		Free	Paid
Test provider	Public Sector	South Korea (RAT): 4th April 2022, RAT testing was used in tandem with PCR testing for those undergoing mandatory quarantine—for foreign nationals on day 6 or 7 or quarantine [90]	
	Private Sector		UK England (RAT): 24th Oct 2021, RATs were enabled to be used for arriving travellers, for purchase from private providers. Widespread issues were recognised with commercial providers of testing kits for travel purposes [160] Australia (RAT): 22 January 2022 onwards, In Queensland, fully vaccinated international travellers were enabled to use rapid antigen tests within 24 h of arrival, whereas unvaccinated travellers still had to attend 14-day quarantine [161] Ireland (N/A): Since 6th March 2022, all travel testing requirements were repealed [162] South Africa (N/A): After June 2022, all travel testing requirements were repealed [163] Spain (N/A): After October 21 st 2022, all travel testing requirements were repealed [93]

The approach taken in South Korean for delivering PCR tests at public clinics and transporting individuals to managed accommodation was organisationally complex and labour intensive [91]. South Korea implemented the use of RATs in tandem with PCR testing for post-arrival screening of travellers undergoing isolation, before border screening requirements were abandoned on October 1 st [90]. The uptake of RATs was common across our study countries, and is similarly a shift from public to private provision was also observed.

Australia’s case rates greatly increased with the Omicron variant’s emergence in December 2021-January 2022 [122]. From April 2022 [164], fully vaccinated individuals were allowed to purchase over-the-counter rapid antigen tests, to release from home quarantine [165]. Similarly, in late-October 2021, the German government ceased provision of free testing for any unvaccinated person, following concerns that publicly-funded tests offered an alternative for vaccine-hesitant individuals [166]. Moreover, from 24th October 2021 in the UK, private companies were allowed to provide RATs, rather than PCR tests, direct to customers for post-arrival testing [160].

The cases studied reflect how changing internal circumstances (including vaccination rates) were associated with counties moving to screening systems that shifted screening costs to individual travellers, while reducing burden on public finances. This mirrors the earlier reliance on private provision and traveller self-funding of diagnostic testing observed for pre-departure testing in these countries. The decision to rely on private provision of screening for arrivals was likely influenced by numerous factors, such as those relating to testing capacity, epidemiology, economic imperatives, and political issues [167]. However, the governmental decision-making process was often not publicly available to scrutinise [168, 169]. Instead, we can compare the challenges that arose following these decisions and the responses governments took, to inform future conceptions of border screening systems.

Increasing rates of vaccination in 2021 and 2022 reduced the risk of importing cases, with the phasing out of border measures such as mandatory quarantine and border screening. Prior to this, nations scaled back their border measures for specific groups such as non-nationals, non-vaccinated individuals, or individuals from specific high-risk countries. This produced a gradual end to border screening measures over the course of 2022 and early 2023.

## Discussion

Border screening for travellers was widely introduced by countries during the COVID-19 pandemic, despite a lack of evidence for its efficacy as a public health measure in the pre-pandemic period [6], stimulating debate on this issue [170]. Many countries deemed border screening necessary to reduce transmission, regardless of the considerable organisational challenges and financial costs of its implementation [62, 109].

### How did countries configure their border screening systems for COVID-19?

With minimal guidance available to steer countries towards a consensus approach we observed considerable variation in the configuration of border screening systems across the study countries.

By applying Lee *et al.*’s [38] dimensions of ‘stage of journey’ and the ‘public–private continuum’ across the border screening systems of eight countries, we find both diversity and consensus in different aspects of screening system configurations. First, we found a strong consensus across all study countries in their reliance on private testing providers for delivery of the pre-departure screening that could prevent their export of cases. This sharply contrasts with how the same countries relied on public sector provision of border screening for arriving inbound travellers, particularly if the study country had maintained low disease prevalence and had a higher incentive to avoid the importation of new cases. However, there was considerable divergence in the configuration of post-arrival screening systems. While some countries centralised PCR testing (e.g. South Korea and Australia) with public provision of screening services, others allowed diverse mixes of public and private providers as well as PCR and RAT provision. This was particularly notable in Germany which changed its federal policy multiple times in our first study period, and devolved administration of screening to states that further diversified policies across the country.

### In what contexts did states rely on public or private providers of these services?

In terms of private provision, as mentioned above, countries did consistently rely on private provision of pre-departure screening, while requiring that this be paid for by travellers. The delegation of responsibility to the private sector for screening of outgoing travellers demonstrates a clear policy priority as national governments took more responsibility for limiting the *importation* of cases as opposed to limiting the *exportation* of cases across their borders, which they devolved to the private sector. However, this would change as new technologies became available and the risks from COVID-19 became more manageable. Demand for border screening grew with resumption of international travel, increasing vaccination rates, and higher prevalence rates of infection associated with the Omicron variant(s). Countries that had thus far relied on public sector provision for post-arrival testing transitioned to enabling private provision, including with less sensitive RATs (as was seen in Australia for example). This reflects a change in the cost–benefit calculus associated with border screening under new epidemiological and technological circumstances; more travellers and therefore more testing required methods of border screening which could cope with growing volumes, without expanding public sector costs.

Our international comparison shows that when using the private sector to reduce the burden on public resources a range of problematic issues arose, around the quality of testing and services, ensuring adherence to standards and requirements as well as appropriate pricing of tests. Reduced reliance on the public sector for screening capacity increased the state’s burden of oversight and reliance on governance bodies to ensure external providers maintained the required focus on public health goals. This observation is particularly relevant in the context of a public health emergency where new diagnostic service providers are entering the market space and testing technologies are novel [67, 68]. The impact of private provision of diagnostic testing on governance and oversight bodies remains an unexplored complexity in the private provision of health services, warranting further study.

Countries that conduct border screening using public health resources faced significant challenges of high resource costs and organisational complexity. However, one rationale for keeping this screening activity under the scope of public provision is quality control for clinical services, oversight of testing services, as well as linkage to other pandemic management systems such as sequencing of variants and isolation enforcement [57, 171–173]. As we have seen, these activities can be hindered when entrusting this activity to the private sector, without adequate oversight.

This is not to say that organising screening through the public sector is always more desirable. A government’s decision to publicly provide border screening may be based on multiple factors, including: the epidemiology of the country, passenger numbers, feasibility of publicly funding screening of all travellers, and other rationales for enabling international travel such as economic incentives by supporting tourism. All of these rationales are measured against the great resource costs to conducting border screening using public funds.

### What do policies and narratives reveal about the perceived role of border screening in global public health?

Our findings contribute to the discussion of health as a foreign policy issue, where a tension is present between the need for cooperation, recognised following the 2003 SARS pandemic [82] and as recommended under the IHR (2005), and the pragmatic politics of international health management [174, 175]. Pre-departure COVID-19 screening was found to not be provided by public health authorities in any of our study countries, but instead was delegated to the private sector. While many discuss this in the context of international travel being a ‘luxury’, [63, 65, 176] this choice can also be seen as delegating responsibility for a measure which could potentially limit case exportation to vulnerable countries, while states instead focused their resources on preventing case importation.

This framing is particularly notable when one considers the IHR obligation of states to pay for screening in contexts where travellers are required to be tested as a condition of entry to a territory (IHR Art. 40, paragraph 1). If international travel is considered to be a ‘luxury’, then the obligation of the state to pay for such screening may be negated because this is not considered a necessity for the states’ own public health.

These implications are further extended when one considers that pre-departure screening requirements were observed to be set not by the country of origin (i.e. where they are travelling *from*), but by the destination country (where they are travelling *to*). By creating requirements for individuals in *other* territories, countries are making policy that is implemented through another country’s provision of screening. In effect, destination countries seek to limit case importation by requiring countries of origin to implement measures that limit case exportation. This demonstrates again the defensive priority embodied in the configuration of border screening. Even where measures are implemented to prevent the *export* of cases, this is only done according to the requirements of the destination countries (rather than altruistically seeking to minimise export of cases through consistently thorough tests), while being implemented through private providers and paid for by individual travellers.

Moreover, the setting of domestic policy which is enacted in another state’s territory may create uncertainty over which state is obliged to pay under IHR Art 40. The article’s text states that “no charge shall be made by a State Party … [for] examination which may be required by required by *that* State Party” which does imply that it is the mandating country’s obligation. However, the wording of the text does not make it clear whether the charge relates specifically to the State Party mandating the test or the policy with respect to *how* the test is provided in the country of origin. Importantly, this meant that many states opted instead to push the responsibility for paying for pre-departure testing onto the individual, while relying on the ‘luxury’ framing of travel.

Comparatively, for countries accepting travellers into their jurisdiction, any public resources invested in post-arrival screening can be expected to provide direct public health benefits to their country (i.e. preventing an increase in diseases prevalence due to case imports). As such, more countries dedicating public resources to these activities emphasises the policy priority of protecting their own populations. This is particularly clear when regarding the evolution of post-arrival screening as the Omicron variants emerged and rapid antigen tests became available. As the volume of travellers increased and the cost of tests decreased, a move to traveller-paid post-arrival screening was observed. However, this was mainly as part of ‘test-to-release’ programmes where travellers could pay for testing which could shorten quarantine periods. In doing so, the testing could again be framed as a ‘luxury’ commodity and primarily for the *traveller’s* benefit, rather than for public health purposes, complying more closely to IHR Art 40, para 2. Requiring that this test now be paid for by the traveller again shifts the costs from the destination country while maintaining the public health benefit; travel testing is further revealed to be oriented towards the prevention of case importation.

## Conclusions

Nations responded to the COVID-19 pandemic with unprecedented levels of border screening, which required massive increases in diagnostic testing capacity. Relying on the private sector to deliver pre-departure screening was a common strategy to achieve the required service volume, however the challenges identified in this international comparative analysis show that public health outcomes are not always prioritised through private provision. This has implications for the cross-border cooperation in diagnostics recommended as part of the current IHR (2005) recommendations. Delegating screening activities to private providers with minimal guidance or standards, can lead to suboptimal quality of service provision, that may reduce the efficacy of those measures. Experiences from the COVID-19 pandemic provide an opportunity to improve cross-border diagnostic testing by understanding and mitigating such challenges.

Our findings also emphasise a key issue central to debates around border measures in global health. The significant lack of international consensus on whether border measures are effective [6], legal [34], or acceptable [9] sits in tension with their consistent and widespread use by states during public health emergencies. For example, as we have shown here, national policy perspectives on border measures prioritise the prevention of case importation despite studies which conclude that they are not effective [3, 4, 6, 9, 177, 178]. However, the focus on preventing case exportation is one which is often advocated in policy (as by WHO in early 2020), and has had some validation of efficacy (e.g. Grepin et al.) [6]. When discussing border measures and their implementation, then, attention should not reside purely with which policies were enacted but also with the intent of those policies (altruistic or defensive) in order to understand their effectiveness.

This paper does not aim to answer definitively whether travel screening is specifically effective or legal, but rather to note that implementation comes in a variety of forms and these evolved throughout the COVID-19 pandemic. These positions suggest a particular, internally-oriented perspective on the prevention of international spread of infectious disease. Further study is required to better understand the efficacy of such measures in preventing global spread of infectious diseases, as well as to better understand the equity implications of such a perspective, given the current unequal distribution of access to health both nationally and internationally.

Moreover, our analysis is based upon publicly available documentation and reportage which naturally limits the extent to which we can definitively infer or evidence individual country or government *motivations* when it comes to these measures. Analysis of the policies and the ways in which they evolved over time provides us with insight into possible motives, but the analytical frame here is consistently on the underlying assumptions and objectives of policies rather than direct attribution of motive and individual trade-offs.

However, initial WHO recommendations for COVID-19 [8] outline border screening as a policy which can decrease case *exportation*, as do the core capacities of the IHRs [39]. This perspective is important because studies in Grépin et al.’s [5] systematic review did find that border measures had some efficacy in preventing case exportation. The possibility of preventing exportation runs counter to how we would expect self-interested nations to act, but sits well with the new health governance that Fidler outlines [12], whereby nation states join a ‘compact’ to protect one another rather than just themselves.

As noted above, international travel hubs often are located within higher-income countries and thus current statements of international norms (i.e. the guiding ethos of the IHR) place the responsibility on these states to limit the *exportation* of cases as a measure for limiting global spread. This is not, however, what our findings demonstrate with respect to the way travel screening was organised during the COVID-19 pandemic. We therefore emphasise a need for wealthier countries to ensure that they are meeting the spirit of their IHR obligations by limiting case exports, rather than focusing so predominantly on addressing case imports.

## Data Availability

All data generated or analysed during this study are included in this published article.

## Abbreviations

COVID-19: Coronavirus 2019, disease caused by the SARS-CoV-2 virus
IHR: International Health Regulations
PCR: Polymerase Chain Reaction
LFT: Lateral Flow Test
RAT: Rapid Antigen Test
PPV: Percentage Positive Value
HCP: Healthcare Professional
NHS: National Health Service
CMA: Competition and Markets Authority
DHSC: Department of Health and Social Care
HIQA: Health Information and Quality Authority
CTM: Corporate Travel Management
UKAS: UK Accreditation Service
UN: United Nations
WEF: World Economic Forum
WHO: World Health Organisation
EU: European Union
